# Machine Learning Algorithms Predict Prolonged Opioid Use in Opioid-Naïve Primary Hip Arthroscopy Patients

**DOI:** 10.5435/JAAOSGlobal-D-21-00093

**Published:** 2021-05-25

**Authors:** Kyle N. Kunze, Evan M. Polce, Thomas D. Alter, Shane J. Nho

**Affiliations:** From the Department of Orthopaedic Surgery, Hospital for Special Surgery, New York, NY (Dr. Kunze); and the Department of Orthopaedic Surgery, Division of Sports Medicine, Rush University Medical Center, Chicago, IL (Mr. Polce, Mr. Alter, and Dr. Nho).

## Abstract

**Introduction:**

Excessive opioid use after orthopaedic surgery procedures remains a concern because it may result in increased morbidity and imposes a financial burden on the healthcare system. The purpose of this study was to develop machine learning algorithms to predict prolonged opioid use after hip arthroscopy in opioid-naïve patients.

**Methods:**

A registry of consecutive hip arthroscopy patients treated by a single fellowship-trained surgeon at one large academic and three community hospitals between January 2012 and January 2017 was queried. All patients were opioid-naïve and therefore had no history of opioid use before surgery. The primary outcome was prolonged postoperative opioid use, defined as patients who requested one or more opioid prescription refills postoperatively. Recursive feature elimination was used to identify the combination of variables that optimized model performance from an initial pool of 17 preoperative features. Five machine learning algorithms (stochastic gradient boosting, random forest, support vector machine, neural network, and elastic-net penalized logistic regression) were trained using 10-fold cross-validation five times and applied to an independent testing set of patients. These algorithms were assessed by calibration, discrimination, Brier score, and decision curve analysis.

**Results:**

A total of 775 patients were included, with 141 (18.2%) requesting and using one or more opioid refills after primary hip arthroscopy. The stochastic gradient boosting model achieved the best performance (c-statistic: 0.75, calibration intercept: −0.02, calibration slope: 0.88, and Brier score: 0.13). The five most important variables in predicting prolonged opioid use were the preoperative modified ones: Harris hip score, age, BMI, preoperative pain level, and worker's compensation status. The final algorithm was incorporated into an open-access web application available here: https://orthoapps.shinyapps.io/HPRG_OpioidUse/.

**Conclusions:**

Machine learning algorithms demonstrated good performance for predicting prolonged opioid use after hip arthroscopy in opioid-naïve patients. External validation of this algorithm is necessary to confirm the predictive ability and performance before use in clinical settings.

US citizens consume approximately 80% of the global opioid supply, and the total economic burden of opioid overdose, abuse, and dependence in the United States has now been estimated to be $78.5 billion.^1,2^ Because pain is inevitable after a surgical intervention, increased criticism surrounding prescribing practices concerning opioid medications has become widely recognized.^3,4^ Therefore, preoperative prediction of prolonged opioid use after surgical procedures could be of great clinical utility by providing an opportunity for targeted intervention and shared decision-making in efforts to avoid such risk.

Several recent studies have sought to provide insight into the factors predicting prolonged opioid use and the implications of opioid dependence after orthopaedic procedures.^5,6,7,8^ Karhade et al^9^ developed machine learning algorithms to predict prolonged opioid use in opioid-naïve lumbar spine surgery patients and found that the best algorithm demonstrated moderate discriminative capacity for identifying at-risk patients. Other studies have applied machine learning and predictive modeling to identify patients of prolonged opioid use after total hip arthroplasty^10^ in addition to surgery for lumbar disk herniation^11^ and anterior cervical diskectomy and fusion.^12^ Concerning hip arthroscopy specifically, Beck et al^13^ recently reported that prolonged opioid use is a risk factor for failing to achieve clinically notable outcome improvement; however, factors predictive of opioid dependence after hip arthroscopy and the potential predictive value of machine learning to this end remain poorly understood.

The purpose of this study was to develop machine learning algorithms to predict prolonged opioid use after hip arthroscopy in opioid-naïve patients. The authors hypothesized that the best of the five machine learning algorithms would provide excellent discriminatory and predictive capabilities, allowing for the development of a web application that could provide patient risk at the individual level.

## Methods

### Guidelines

The Transparent Reporting of a multivariable prediction model for Individual Prognosis or Diagnosis guidelines and the Guidelines for Developing and Reporting Machine Learning Models in Biomedical Research were adhered to in the present analysis.^14,15^

### Patient Selection and Data Source

Institutional review board approval was obtained to query a clinical database of secure, prospectively collected data from a dedicated hip preservation center. The inclusion criteria for this study consisted of all patients undergoing primary hip arthroscopy for femoroacetabular impingement syndrome between January 2012 and January 2017. Exclusion criteria included (1) revision hip arthroscopy, (2) history of congenital hip pathology (developmental dysplasia of the hip, slipped capital femoral epiphysis, and Legg-Calve-Perthes), and (3) previous opioid use.

### Primary Outcome

The primary outcome prolonged opioid use after hip arthroscopy. The postoperative pain protocol for all hip arthroscopy patients included 1 g of oral tylenol every 8 hours, 15 mg of oral meloxicam every 12 hours as needed, 20 pills of oral tramadol 50 mg every 6 hours as needed, and 30 pills of oral oxycodone 5 mg every 4 hours as needed. Prolonged opioid use was therefore defined as patients who requested, filled, and used some or all of an additional opioid prescription.

### Candidate Predictor Variables and Handling of Missing Data

Potential variables for prediction consisted of a total of 17 candidate variables that were collected prospectively in a secure clinical repository before the index hip arthroscopy procedure (Table 1). No variable was excluded for exceeding greater than 30% of missing data and therefore was considered appropriate for imputation and testing the predictive value.^16,17^ Before analysis, missingness of data was explored and determined to be missing at random and appropriate for multiple imputations. Missing data were adjusted for by using multiple imputations and predictive mean matching with the “mice” package in R (R Foundation for Statistical Computing).^18^ After imputation, recursive feature elimination with random forest algorithms was used to determine the combination of variables with the highest predictive value that optimized algorithm performance through a process of backward elimination.^19,20^

**Table 1:** Baseline Demographic Information of Study Population (n = 775)

Characteristic	Median (IQR) | No. (%)	Rates of Missing Data | No. (%)
Age, yrs	34.0 (23.0-44.0)	0 (0%)
Body mass index, kg/m^2^	24.3 (21.9-27.8)	0 (0%)
Female sex	523 (67.5%)	
Race		83 (10.7%)
White	605 (87.4%)	
Workers compensation	35 (4.7%)	27 (3.5%),
Drug allergies	219 (31.4%)	78 (10.1%)
Sports participation	539 (73.9%)	46 (5.9%)
Smoking	79 (10.4%)	14 (1.8%)
Back pain	103 (13.5%)	14 (1.8%)
Spine pathology	150 (27.3%)	226 (29.2%)
Anxiety or depression	82 (14.1%)	194 (25.0%)
Symptom duration > 2 years	148 (21.2%)	77 (9.9%)
Alpha angle on AP radiograph, degrees	59.0 (54.0-65.0)	79 (10.2%)
LCEA, degrees	32.0 (28.8-36.0)	75 (9.7%)
VAS for pain	70.0 (50.0-80.0)	207 (26.7%)
Preoperative mHHS	59.0 (50.0-68.0)	227 (29.3%)
Preoperative HOS-ADL	66.2 (52.5-79.1)	193 (24.9%)
Preoperative HOS-SS	39.3 (25.0-56.3)	184 (23.7%)
Opioid refill	141 (18.2%)	0 (0%)

AP = anterior-posterior, HOS-ADL = Hip Outcome Score—Activities of Daily Living, HOS-SS = Hip Outcome Score—Sports Subscale, IQR = interquartile range, LCEA = lateral center edge angle, mHHS = modified Harris hip score, VAS = visual analog scale

### Development of Algorithms

The study population (n = 775) was divided using an 80:20 stratified split into training (n = 621) and testing (n = 154) cohorts. The combination of variables identified through recursive feature selections was used to create machine learning algorithms for training. Algorithms were subsequently trained using 10-fold cross-validation repeated five times. The following algorithms were developed on the training set: (1) stochastic gradient boosting, (2) random forest, (3) support vector machine, (4) neural network, and (5) elastic-net penalized logistic regression. After algorithm development and training, the performance of algorithms was tested and internally validated on the independent (hold-out) set of patients who had not been seen by the algorithms before.

### Algorithm Performance Assessment

Metrics used for model performance assessment were discrimination (area under the receiver operating characteristic curve), calibration (calibration plot, intercept, and slope), Brier score, and decision curve analysis.

For discrimination, receiver operator curve analysis was used to calculate a concordance statistic (c-statistic). The c-statistic in this study describes the probability that the model assigns a greater predicted probability to a randomly selected positive case (prolonged opioid use) relative to a randomly selected negative case (no prolonged opioid use).

Calibration assesses the relationship between predictive probabilities of the model and observed frequencies of events (prolonged opioid use) in the study population. The calibration intercept represents the propensity of the model on an average to overestimate or underestimate the observed outcome prevalence, whereas the calibration slope reflects whether predictions were precise or too extreme.^21,22^ A model with perfect predictive capabilities has a calibration slope = 1 and calibration intercept = 0.

The Brier score reflects both calibration and discrimination and describes the overall performance of the model.^23^ The Brier score is determined by calculating the mean squared difference between true outcomes and the corresponding predicted probabilities from the model.^24,25^ The null model Brier score (assigning a probability of prolonged opioid use equivalent to the prevalence of prolonged opioid use) was calculated to benchmark the algorithm performance.

Decision curve analysis allows for extrapolation of clinical utility of each model by comparing the predicted net benefit at varying risk thresholds.^22^ Curves that remain at higher values on the y-axis with increasing risk thresholds indicate that following that particular management decision (in this case, choosing to perform hip arthroscopy based on the machine learning model) will confer greater benefit (where benefit is a low probability of opioid dependence) to patients than other management decisions.

### Algorithm Fidelity Assessment and Open-source Application Development

Local model behavior was explored through local interpretable model-agnostic explanations (LIMEs).^26^ LIME is a visualization technique that provides insight into the decision-making of complex “black box” models by training separate interpretable models to explain the local behavior of the model and how it comes to a prediction (what specific combination of factors supported or contradicted a specific patient to experience postoperative opioid dependence) and explain how the model came to a decision regarding a prediction. The best performing algorithm, LIME, was used to create a patient-specific prediction application in a digital format. This open access digital web application was developed with the capacity to provide both predictions and explanations at the individual patient level. Data analysis was performed using R (The R Foundation) and RStudio.

## Results

### Study Population

Overall, 775 patients met the inclusion criteria and had a median age of 34 (interquartile range 23.0 to 44.0) years. A total of 523 (67.5%) patients were woman (Table 1). A total of 219 (29.3%) patients were overweight (body mass index [BMI] 25 to 29.9), and a total of 107 (13.8%) were obese (BMI ≥ 30). The incidence of one or more opioid refills in opioid-naïve patients after primary hip arthroscopy was 141 (18.2%). The mean number of additional opioid prescription refills was 1.43 (range, 1 to 6).

### Feature Selection

The following variables were identified through recursive feature selection with random forest algorithms for prediction of prolonged opioid use after hip arthroscopy: the preoperative modified Harris hip score (mHHS), age, BMI, preoperative visual analog scale (VAS) pain score, workers compensation status, sports participation, and race.

### Algorithm Performance

**Figure 1:**
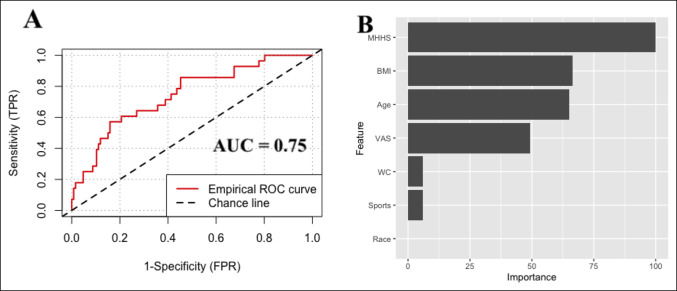
Graph showing the (**A**) discrimination of the stochastic boosting gradient algorithm and (**B**) relative variable importance, testing set (n = 154). The relative variable importance plot shows the relative importance of variables used for predicted indexed against the most important variable (mHHS). BMI = body mass index, mHHS = modified Harris hip score, VAS = visual analog scale

Internal validation of the five algorithms on the independent testing set (n = 154) resulted in c-statistic ranging from 0.62 to 0.75, calibration intercept ranging from −0.11 to 0.11, calibration slope ranging from 0.37 to 1.06, and Brier score ranging from 0.13 to 0.15. The null model Brier score was 0.16 (Table 2). The algorithm with the best performance was the stochastic boosting gradient with area under the curve (AUC) 0.75 (Figure 1, A), calibration intercept −0.02, calibration slope 0.88, and Brier score 0.13. Global variable importance modeling demonstrated that the five most important features for predicting prolonged opioid use were preoperative mHHS, age, BMI, preoperative VAS pain, and workers compensation status (Figure 1, B).

**Table 2:** Algorithm Performance in Independent Testing Set (95% Confidence Interval), n = 154

Metric	Stochastic Gradient Boosting	Random Forest	Support Vector Machine	Neural Network	Elastic-net Penalized Logistic Regression
C-statistic	0.75 (0.63 to 0.83)	0.73 (0.62 to 0.80)	0.62 (0.47 to 0.72)	0.74 (0.63 to 0.83)	0.75 (0.65 to 0.83)
Calibration intercept	−0.02 (−0.47 to 0.43)	0.11 (0.34 to 1.15)	−0.11 (−0.54 to 0.32)	0.09 (−0.36 to 0.54)	−0.07 (−0.50 to 0.37)
Calibration slope	0.88 (0.46 to 1.30)	0.74 (0.34 to 1.15)	0.37 (−0.22 to 0.95)	0.67 (0.28 to 1.06)	1.06 (0.51 to 1.60)
Brier score	0.13 (0.093 to 0.16)	0.14 (0.10 to 0.17)	0.16 (0.12 to 0.20)	0.14 (0.10 to 0.18)	0.13 (0.09 to 0.17)

Null model Brier score = 0.16.

**Figure 2:**
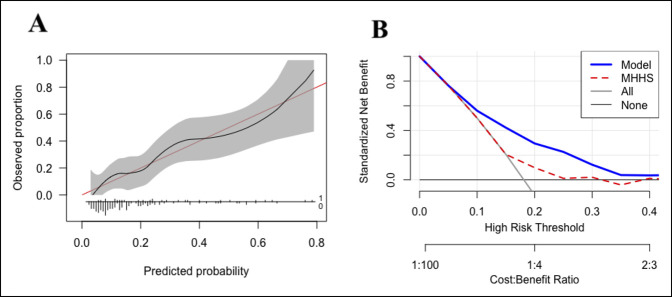
Line graph showing (**A**) calibration and (**B**) decision curve analysis of the stochastic boosting gradient algorithm, testing set (n = 154). Net benefit is displayed on the *y*-axis, whereas the predicted probabilities are plotted along the *x*-axis. In this analysis, the net benefit is defined as the weighted average of true positives and false positives generated by the model at a given threshold. The utility of the model is compared with changing management for all patients who present for hip arthroscopy, no patients who present for hip arthroscopy, based on the most important feature alone (mHHS), and based on the model. mHHS = modified Harris hip score

Calibration analysis demonstrated that the model predicted the probability of prolonged opioid use in close approximation with the observed distribution without overfitting (Figure 2, A). Decision curve analysis of the stochastic boosting gradient showed that the algorithm resulted in greater net clinical benefit than the default strategies of changes for all patients, for no patients, or changes based on preoperative mHHS alone (Figure 2, B).

### Real-world Application Utility

**Figure 3:**
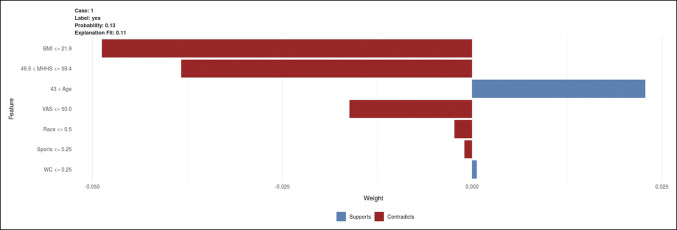
Graph showing an example of individual patient-level explanation for stochastic boosting gradient predictions. The patient-specific risk of prolonged opioid use in this scenario is 13.0%. In this scenario, body mass index, a modified Harris hip score between 49.5 and 59.4, low preoperative pain (VAS less than 50), and White race (0 = White, 1 = other, and therefore <0.5 is a proxy for equal to 0) decrease the risk of prolonged opioid use. Age greater than 43 years supports the risk of needing an opioid refill, whereas no sports participation (equivalent to 0) and no workers compensation claim (equivalent to 0) have marginal effects for this particular patient. mHHS = modified Harris hip score, VAS = visual analog score

The final algorithm was transformed into an interactive interface and deployed as an open access digital application accessible on desktops, tablets, and smartphones. An example of the individual patient-specific explanations for a theoretical patient scenario using this application is shown in Figure 3. For each patient and individual risk assessment, the LIME plot is generated, depicting which aspects of their history support (increase the risk of opioid dependence) or contradict (decrease the risk of opioid dependence) the prediction. Because it currently stands, these algorithms should not be used in clinical settings; rather, they serve as an educational tool and demonstrate the power of machine learning.

## Discussion

The main findings of this study are as follows: (1) the stochastic gradient boosting was the best performing machine learning algorithm; (2) the five most important features for predicting opioid dependence in this population were the preoperative mHHS, age, BMI, preoperative pain level, and worker's compensation status; and (3) the stochastic gradient boosting model was incorporated into an open-access web application that may have clinical utility after rigorous external validation and confirmation of its predictive ability.

The best performing machine learning algorithm in this study showed good discriminatory ability and used critical model metrics of assessment for machine learning to demonstrate the extent of predictive capabilities. The performance of the best algorithm in this study (c-statistic = 0.75) is comparable with other machine learning studies, which have sought to predict prolonged opioid use in other areas of orthopaedics. Karhade et al^9^ found that their best machine learning algorithm had a c-statistic of 0.70 for predicting prolonged opioid use in opioid-naïve patients after lumbar spine surgery. Similarly, Karhade et al reported that their best algorithm, the elastic-net penalized logistic regression, achieved a c-statistic of 0.77 for predicting prolonged opioid use after total hip arthroplasty. Despite being the first study to our knowledge to apply these methods of machine learning to a hip arthroscopy population, the similarities in performance of the stochastic boosting gradient validates the potential utility of these methods in such patients.

The features selected by recursive feature elimination are in accordance with predictors of prolonged opioid use in previous orthopaedic studies. Indeed, age has been found to be a strong predictor of prolonged opioid use in total hip arthroplasty patients.^10,27^ Both Gil et al^28^ and Nicholson et al^29^ reported that insurance and compensation category conferred and increased odds of prolonged opioid use in opioid-naïve patients undergoing shoulder surgery, whereas Mohamadi et al^30^ found this same relationship for patients undergoing orthopaedic trauma surgery in a recent meta-analysis. Yang and Werner^31^ found that BMI was markedly associated with an increased likelihood of prolonged opioid use after spinal fusion for idiopathic scoliosis, whereas Nicholson also corroborated this finding for shoulder surgery patients.^29^ Hadlandsmith et al^32^ found that race was markedly associated with prolonged opioid use in patients undergoing total knee arthroplasty, whereas Namba et al^33^ found that both race and preoperative pain levels were risk factors for prolonged opioid use after total knee arthroplasty.

Because pain level contributes to approximately one-half of the scoring criteria for the modified Harris hip score, it is plausible that this outcome measure, which also includes function, was found to be highly predictive of prolonged opioid use in this population. In addition, this study found that the preoperative VAS pain was an important predictor of prolonged opioid use. Therefore, importantly, other patient-reported outcome measures with similar weighting for pain may have some predictive value for opioid use, although these were not investigated in this study because they were not routinely collected. Regardless, this information is critical for hip arthroscopists because prolonged postoperative opioid use has been associated with a higher risk of revision hip arthroscopy and conversion to total hip arthroplasty.^8^ Moreover, the validation of such risk factors may be particularly useful in contributing to decreasing the incidence of prolonged opioid use in this particular field of orthopaedic surgery.

This study may have notable clinical implications in the context of nationwide opioid concerns, given that it is the first machine learning study for prolonged opioid use in elective hip arthroscopy. Furthermore, this study deployed machine learning algorithms as an open-access application, which after external validation may be integrated into electronic medical records to augment shared decision-making between hip arthroscopists and new patients. By better understanding the preoperative patient-specific factors contributing to opioid dependence, providers can ultimately work with patients through discussion of their specific risk factors and potentially directing them toward a health optimization pathway (ie, weight loss, improvement of function through therapy, and improving the mHHS) to decrease their risk of opioid dependence. However, we currently do not support the use of this application in clinical practice because they require rigorous external validation. The purpose of creating an online application is educational and intended to demonstrate the real-world applications of machine learning.

The following limitations should be considered in the context of the current study results. Although the best performing algorithm was rigorously tested through four different assessments, was internally validated, and demonstrated good predictive ability, external validation was not possible. Determining the performance of this model through external validation will be essential before widespread adoption of this tool. Furthermore, it is not possible to test all of the potential variables that may contribute to opioid dependence after hip arthroscopy despite using a large pool of covariates that are routinely collected at our institution. Possibly, other factors not routinely collected at our institution may have some contributions to the potential for opioid dependence in opioid-naïve patients.

## Conclusion

Machine learning algorithms demonstrated good performance for predicting prolonged opioid use after hip arthroscopy in opioid-naïve patients. External validation of this algorithm is necessary to confirm the predictive ability and performance before use in clinical settings.
